# Nuclear factor I-C regulates E-cadherin via control of KLF4 in breast cancer

**DOI:** 10.1186/s12885-015-1118-z

**Published:** 2015-03-10

**Authors:** Hye-Kyung Lee, Dong-Seol Lee, Joo-Cheol Park

**Affiliations:** Department of Oral Histology-Developmental Biology & Dental Research Institute, School of Dentistry, Seoul National University, 101 Daehagro, Chongro-gu, Seoul, 110-749 South Korea

**Keywords:** NFI-C, KLF4, E-cadherin, Tumorigenesis

## Abstract

**Background:**

Progression to metastasis is the leading cause of most cancer-related mortality; however, much remains to be understood about what facilitates the spread of tumor cells. In the present study, we describe a novel pathway in breast cancer that regulates epithelial-to-mesenchymal transition (EMT), motility, and invasiveness.

**Methods:**

We examined nuclear factor I-C (NFI-C) expression in MCF10A human breast epithelial cells, MCF7 non-invasive breast cancer cells, and MDA-MB231 invasive breast cancer cells by real-time PCR and western blotting. To investigate the loss- and gain-function of NFI-C, we determined whether NFI-C regulated KLF4 expression by real-time PCR, western blotting, and promoter assay. To understand the biological functions of NFI-C, we observed cell invasion, migration, adhesion in human tumor cells by transwell assay, wound healing assay, quantitative RT-PCR, cell adhesion assay, western blotting, and immunohistochemistry.

**Results:**

We identified the downstream factors of NFI-C, such as KLF4 and E-cadherin, which play roles in EMT. NFI-C is expressed in normal mammary gland or noninvasive breast cancer cells with epithelial characteristics. NFI-C overexpression induced expression of KLF4 and E-cadherin, but not Slug, in breast cancer cells. NFI-C bound directly to the KLF4 promoter and stimulated KLF4 transcriptional activity, thereby regulating E-cadherin expression during tumorigenesis. Cells overexpressing NFI-C maintained their epithelial differentiation status, which could drive mesenchymal-epithelial transition (MET) via the NFI-C-KLF4-E-cadherin axis in breast cancer cells. Consequently, NFI-C suppressed EMT, migration, and invasion in breast cancer cells.

**Conclusions:**

Our study reveals a novel signaling pathway that is important during breast cancer tumorigenesis: the NFI-C-KLF4-E-cadherin pathway. The results indicate the important role of NFI-C in regulating KLF4 during tumorigenesis.

## Background

The roles of Krüppel-like factor 4 (KLF4) have been studied in many physiological processes, including development, cytodifferentiation, and maintenance of normal tissue homeostasis [1]. KLF4 is a zinc-finger transcription factor which is usually expressed in growth-arrested cells and differentiated cells of the colon, small intestine, lung and testis [2]. Also, the expression of KLF4 is downregulated in several cancer types [3-5]. Database analysis reveals a correlation between low KLF4 expression and an increased incidence of malignant breast carcinoma [6]. KLF4 functions as both a transcriptional activator and a repressor at various promoters in a context-dependent manner. For example, KLF4 acts as a tumor suppressor by binding to and repressing *p53* promoters but activating the promoter of *p21*, a gene involved in cell cycle inhibition [7]. Interestingly, together with Oct4, Sox2 and c-Myc, KLF4 is a pivotal factor in the generation of induced pluripotent cells and is required for the epigenetic reprogramming of a somatic genome [8,9]. KLF4 is necessary to maintain the proper morphology of epithelial cells. While loss of KLF4 function induces EMT-like morphological changes, forced expression of KLF4 in invasive breast cancer cells induces epithelial differentiation by directly repressing the expression of Snail1, a potent repressor of E-cadherin gene expression, and directly binding to the *E-cadherin* promoter and upregulating E-cadherin expression [10].

The nuclear factor I (NFI) family of site-specific transcription factors, encoded by four genes in vertebrates (termed *NFI-A*, *NFI-B*, *NFI-C*, and *NFI-X*), plays essential developmental roles in the transcriptional modulation of various cell types [11]. In mammary epithelial cells, NFI-C has an important role in prolactin signaling with Jak2, which is independent of the signal transducers and activators of transcription (Stat) pathway [12]. NFI-C activates p53 and participates in the establishment of milk protein gene expression during pregnancy [13]. Interestingly, NFI-C expression indicates better prognosis in breast cancer patients, because it is more highly expressed in normal glandular cells and virtually absent from lymph node metastases. It abolishes tumorigenicity, suppresses EMT, and directly represses *Forkhead box F1* (*FoxF1*), a potent inducer of EMT, invasiveness, and tumorigenicity [14,15]. Our previous report demonstrated that the crosstalk between NFI-C and TGF-β1 signaling regulated cell differentiation in odontoblasts [16]. Also, NFI-C regulates E-cadherin expression via control of KLF4 during dentinogenesis [17]. However, the precise functions of NFI-C in EMT/MET and tumorigenesis remain largely unknown.

Mesenchymal-epithelial transition (MET) events are defined as those in which mesenchymal cells lose their motile, migratory properties and multipolar or spindle-shaped morphology and acquire cell polarity and adhesion to become planar arrays of polarized cells called epithelium. Both MET and epithelial-mesenchymal transition (EMT) occur in normal tissues, including gastrulating and regenerating tissue, as well as abnormal tissues of fibrotic organs or tumors [18]. Indeed, EMT drives mammary epithelial cells to de-differentiate into mammary stem cells and cancer stem cells, which are mesenchymal-like [19]. Thus, it is necessary to examine the relationship between NFI-C and EMT/MET in that NFI-C plays an antagonistic role of TGF-β1 signaling [16] and controls KLF4 and E-cadherin [17].

Prior to this study, NFI-C were established as important regulators of KLF4, but the relationship between NFI-C and KLF4 during tumorigenesis remained unclear. In the present study, we further investigated the roles of NFI-C and KLF4 and their relationship during tumorigenesis.

## Methods

### Cell culture

All experiments involving human cell lines were performed according to the Dental Research Institute guidelines and the Institutional Animal Care and Use Committees of Seoul National University (SNU-111013-3). MCF7 cells (ATCC, Rockville, MD) were grown and maintained in DMEM (Gibco BRL, Carlsbad, CA) supplemented with 10% FBS and antibiotics in a 5% CO_2_ atmosphere at 37°C. The immortalized human mammary epithelial cell line, MCF10A (ATCC), was cultured in complete MCF10A growth media, composed of DMEM/nutrient mixture F12 (DMEM/F12, Gibco BRL) supplemented with 5% fetal calf serum, 20 ng/ml EGF, 10 mg/ml insulin, 0.5 mg/ml hydrocortisone, and 100 ng/ml cholera toxin (Sigma-Aldrich, St-Quentin Fallavier, France).

### TGF-β stimulation

MCF10A or MCF7 cells were stimulated with TGF-β (10 ng/ml, Invitrogen, Carlsbad, CA) at 37°C for 1 hr.

### Plasmid constructs

The pCH-nuclear factor I-C *(NFI-C)* expression plasmid was provided by Dr. R. M. Gronostajski (State University of New York, Buffalo, Buffalo, NY). siRNAs were synthesized (Integrated DNA Technologies, San Diego, CA) based on 19 nucleotides of *NFI-C* (5′-CCG GTG AAG AAG ACA GAG A-3′) and these siRNA plasmids were prepared using the pSUPER-retro-neo-GFP retro virus siRNA expression vector (OligoEngine, Seattle, WA) according to the manufacturer’s instructions. *KLF4* cDNAs were amplified by PCR and subcloned into Flag-tagged pMXs (Cell Biolabs, San Diego, CA). The pGL2-*KLF4* and -*E-cadherin* plasmids were purchased from Origene (Rockville, MD).

### Real-time PCR analysis

Total RNA was extracted from MCF10A and MCF7 cells as well as pulp tissue using TRIzol® reagent according to the manufacturer’s instructions (Invitrogen). Total RNA (2 μg) was reverse transcribed for 1 h at 50°C with 0.5 mg Oligo dT and 1 μl (50 IU) Superscript III enzyme (Invitrogen) in a 20 μl reaction. One microliter of the RT product was PCR amplified using the primer pairs. For real-time PCR, the specific primers for *NFI-C*, *Slug*, *KLF4*, *Vimentin*, *E-cadherin*, *N-cadherin,* and *P-cadherin* are listed in Table 1. Real-time PCR was performed on an ABI PRISM 7500 sequence detection system (Applied Biosystems, Carlsbad, CA) using SYBR GREEN PCR Master Mix (Takara Bio Inc., Otsu, Shiga, Japan) according to the manufacturer’s instructions. PCR conditions were 95°C for 1 min, 94°C for 15 sec, and 60°C for 34 sec for 40 cycles. All reactions were run in triplicate and were normalized to the housekeeping gene *GAPDH*. Relative differences in PCR results were calculated using the comparative cycle threshold (CT) method.

**Table 1:** **Real-time PCR primer sequences**

Gene name		Primer
hNFI-C	forward	5′-CGA CTT CCA GGA GAG CTT TG-3′
	reverse	5′-GTT CAG GTC GTA TGC CAG GT-3′
hKLF4	forward	5′-CCC ACA CAG GTG AGA AAC CT-3′
	reverse	5′-TTC TGG CAG TGT GGG TCA TA-3′
hSlug	forward	5′-GAG CAT TTG CAG ACA GGT CA-3′
	reverse	5′-CCT CAT GTT TGT GCA GGA GA-3′
hE-cadherin	forward	5′-TGC CCA GAA AAT GAA AAA GG-3′
	reverse	5′-GTG TAT GTG GCA ATG CGT TC-3′
hVimentin	forward	5′-AAA GCG TGG CTG CCA AGA AC-3′
	reverse	5′-GTG ACT GCA CCT GTC TCC GGT A-3′
hN-cadherin	forward	5′-CGA ATG GAT GAA AGA CCC ATC C-3′
	reverse	5′-GGA GCC ACT GCC TTC ATA GTC AA-3′
hP-cadherin	forward	5′-GCA GAA GTC AGC GAG AAA GGA G-3′
	reverse	5′-GGA GGA TGA AAC CAC CCT TCC A-3′
hGAPDH	forward	5′- CCA TGG AGA AGG CTG GGG-3′
	reverse	5′- CAA AGT TCT CAT GGA TGA CC-3′

### Western blot analysis

To prepare whole cell extracts, cells were washed three times with PBS, scraped into 1.5 ml tubes, and pelleted by centrifugation at 12,000 rpm for 2 min at 4°C. After removal of the supernatant, pellets were suspended in lysis buffer [50 mM Tris-Cl (pH 7.4), 150 mM NaCl, 1% NP-40, 2 mM EDTA (pH 7.4)] and incubated for 15 min on ice. Cell debris was removed by centrifugation. Proteins (30 μg) were separated by 10% SDS-PAGE and transferred to nitrocellulose membranes (Schleicher & Schuell BioScience, Dassel, Germany). Membranes were blocked for 1 hr with 5% nonfat dry milk in PBS containing 0.1% Tween 20 (PBS-T) and incubated overnight at 4°C with the primary antibody diluted in PBS-T buffer (1:1000). Rabbit polyclonal anti-NFI-C antibody was produced as described previously [20]. The mouse monoclonal anti-HA (MMS-101P) antibody was purchased from COVANCE (Emeryville, CA). Other antibodies, including those against E-cadherin (sc-7870), N-cadherin (sc-7939), Slug (sc-1539), and GAPDH (sc-25778), were purchased from Santa Cruz Biotechnology (Santa Cruz Biotechnology, CA). The rabbit polyclonal anti-E-cadherin (3195) antibody was purchased from Cell Signaling Technology (Danvers, MA). After washing, membranes were incubated for 1 hr with anti-mouse (sc-2031), anti-rabbit (sc-2004), or anti-goat (sc-2768) IgG secondary antibodies conjugated to horseradish peroxidase (Santa Cruz Biotechnology). Labeled protein bands were detected using an enhanced chemiluminescence system (Dogen, Cambridge, MA). The quantification analyses were performed using Image J (http://imagej.nih.gov/ij/).

### Transient transfection and luciferase assays

MCF7 cells were seeded in 12-well culture plates at a density of 1.5 × 10^5^ cells per well. Cells were transiently transfected with reporter constructs using Metafectene PRO reagent (Biontex, Planegg, Martinsried, Germany). pGL2-*KLF4* or -*E-cadherin* was transfected into cells with *NFI-A-*, *NFI-B-*, *NFI-C-*, *NFI-X-*, *KLF4-*expressing constructs, or the *NFI-C*-siRNA construct. Following the addition of luciferin (50 μl) to the cell lysate (50 μl), luciferase activity was determined using an analytical luminescence luminometer according to the manufacturer’s instructions (Promega, Madison, WI).

### Chromatin Immunoprecipitation (ChIP) assays

After transfection with the indicated plasmid DNA using the metafectene Pro reagent (Biontex), MDPC-23 cells were treated with formaldehyde (1% final concentration) for 10 min at 37°C, rinsed twice with cold PBS, and swollen on ice in lysis buffer [1% SDS, 10 mM EDTA, 50 mM Tris–HCl (pH 8.1)] for 10 min. Nuclei were collected and sonicated on ice. Supernatants were obtained by centrifugation for 10 min and were diluted 10-fold in ChIP dilution buffer [0.01% SDS, 1.1% Triton X-100, 1.2 mM EDTA, 16.7 mM Tris–HCl (pH 8.1), and 167 mM NaCl]. The fragmented chromatin mixture was incubated for 4 h with anti-NFI-C antibody on a rotator at 4°C. Protein A/G PLUS-agarose (30 μl; sc-2003, Santa Cruz) was added and incubated at 4°C for 1 h with rotation to collect the antibody/chromatin complex. The precipitated chromatin complexes were recovered and reversed according to the manufacturer’s protocol (Upstate Biotechnology, Lake Placid, NY). The final DNA pellets were recovered and analyzed by PCR using the specific primers for *Klf4* (612 bp) promoter region: forward, 5′-CTTAGAGAAATAAAAGTAAAGCAGA-3′ and reverse, 5′-TTAGGTTTCCTCAGAATATTTGTGA-3′. The following PCR conditions were used: 94°C for 30 s; 55°C for 30 s; and 72°C for 1 min for 35 cycles. PCR products were electrophoresed in 1% agarose gels, stained with ethidium bromide, and visualized under ultraviolet light.

### DNA affinity precipitation (DNAP) assays

Transfected MCF7 cells were washed with ice-cold PBS, collected by centrifugation, and resuspended in RIPA buffer (50 mM Tris-Cl [pH 7.5], 150 mM NaCl, 1% Nonidet P-40, 1 mM EDTA, 1 mM PMSF, 1 mM Na_3_VO_4_, and 1 mM NaF) supplemented with protease inhibitors (Roche Molecular Biochemicals, Mannheim, Germany). Lysates were rotated on a rotating platform for 30 min at 4°C and purified by centrifugation at 13000 rpm for 5 min at 4°C. Binding assays were performed by mixing nuclear extract proteins (600 μg) and biotinylated specific wild type or mutated NFI-C binding site oligonucleotides (6 μg) [17] of KLF4 promoter in binding buffer (12% glycerol, 12 mM HEPES-NaOH [pH 7.9], 4 mM Tris-Cl [pH 7.9], 60 mM KCl, 1 mM EDTA, 1 mM DTT). Mutated positions in the sequence are underlined [17]. Lysates were incubated at room temperature for 30 min. Next, 60 μl of streptavidin-agarose beads (Thermo Scientific, Rockford, IL) were added. The mixture was incubated for 2 h at 4°C with rotating. Beads were pelleted and washed three times with PBS. NFI-C (wild type or mutant forms) bound to the oligonucleotides was detected by SDS-PAGE and immunoblotting using the mouse monoclonal anti-NFI-C antibody.

### Adhesion assays

Stable cells expressing *NFI-C* or *NFI-C*-siRNA were seeded in 96-well plates and incubated for 4 hr. At the indicated times, plates were washed twice with PBS. Cells were fixed with 4% paraformaldehyde for 30 min, stained with crystal violet for 10 min, followed by the addition of Tween 20 for 30 min. Finally, we measured the OD at 595 nm.

### Wound healing assays

After 24 hr of *NFI-C* or *NFI-C*-siRNA transfection or TGF-β treatment, cells were harvested, seeded in 6-well plates, and cultured until confluent. We used 200 μl pipette tips to make a straight scratch, simulating a wound. Cells were rinsed gently with PBS and cultured in fresh complete media. Cells were imaged with a 10x objective on a Leica DMLB microscope and acquired using QCapture Software (QImaging Software).

### Invasion assays

Cell migration was analyzed using Transwell assays (Corning Inc., Corning, NY) with polycarbonate filters (pore size, 8 μm). Cells transfected with *NFI-C* or *NFI-C*-siRNA or treated with TGF-β were seeded in the upper chamber at a density of 1 × 10^5^ cells/chamber in 100 μl media. The lower chamber was filled with 600 μl DMEM. Plates were incubated for 24 hr at 37°C, and cells in the upper chamber were carefully removed using a cotton swab. Migrated cells were fixed with 4% paraformaldehyde and stained with Hoechst trihydrochloride (1:5000; Invitrogen) for 10 min. The number of invading cells was counted using fluorescent microscopy. Four fields were randomly chosen, and the number of penetrated cells was counted.

### Gene expression omnibus (GEO)-database analysis

Gene expression profile data (GSE2429) of atypical ductal hyperplasia with or without breast cancer cells was downloaded from the National Center for Biotechnology Information Gene Expression Omnibus (NCBI GEO) database (http://www.ncbi.nlm.nih.gov/geo/). Only five pairs of samples are available, one is derived from cancer cells, whereas the other is derived from normal cells, and each pair of samples represents eight breast cancer patients.

### Tissue preparation and immunohistochemistry

The experimental protocol was also approved by the Seoul National University’s Institutional Review Board (S-D2011001). Malignant human breast tissues were obtained retrospectively from the Seoul National University Hospital archive (Seoul, Korea). Human breast tissues were embedded in paraffin, and processed for immunohistochemistry. Sections were incubated overnight at 4°C with rabbit polyclonal NFI-C and E-cadherin as the primary antibodies (dilutions of 1:100–1:200). Secondary anti-rabbit IgG antibodies were added to the sections for 30 min at room temperature, and then reacted with the avidin-biotin-peroxidase complex (Vector Laboratories, Burlingame, CA). Signals were converted using a diaminobenzidine kit (Vector Laboratories). Nuclei were stained with hematoxylin.

### Statistical analysis

Statistical analyses were performed using Student’s t-tests. Statistical significance is denoted by ^*^P < 0.01. All statistical analyses were performed using SPSS software version 19.0 (IBM Corporation, Armonk, NY).

## Results

### NFI-C is expressed in normal epithelial cells, and its expression is reduced during EMT

**Figure 1:**
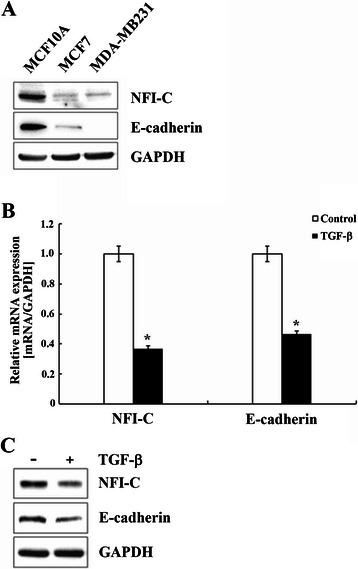
**TGF-**β**-mediated expression of NFI-C and E-cadherin. (A)** Expression of NFI-C and E-cadherin in MCF10A human breast epithelial cells, MCF7 non-invasive breast cancer cells, and MDA-MB231 invasive breast cancer cells analyzed by western blotting. **(B, C)** Expression of NFI-C and E-cadherin in MCF10A cells treated with TGF-β and control MCF10A cells analyzed by real-time PCR **(B)** and western blotting **(C)**.

During tumor progression, carcinoma cells undergo EMT, a process in which polarized epithelial cells undergo dynamic changes to lose their epithelial characteristics and obtain a more motile fibroblastic phenotype, enabling them to proceed with invasion and metastasis [21]. To assess whether the presence of NFI-C in breast tumors has any physiologic relevance, we examined the NFI-C and E-cadherin protein expression in normal human breast epithelial cells, MCF10A, non-invasive breast cancer cells, MCF7, and invasive breast cancer cells, MDA-MB231 by western blotting. NFI-C expression correlated with cellular phenotype. Normal epithelial cells showed high expression of NFI-C and E-cadherin protein; whereas, the noninvasive and invasive cancer cells displayed little to no detectable expression of these two proteins (Figure 1A).

During mammary tumorigenesis, TGF-β signaling plays an important role in EMT by facilitating the change from cuboidal epithelial cells to elongated spindle-shaped cells and causing decreased expression of epithelial markers and enhanced expression of mesenchymal markers [22,23]. Based on the known roles of TGF-β during EMT in MCF10A epithelial cells, we asked whether NFI-C expression is changed during EMT in these cells. Upon TGF-β-induced EMT, a significant reduction in the level of NFI-C and the epithelial marker E-cadherin indicated that these cells lost some of their acquired epithelial features (Figure 1B and C).

NFI-C maintains epithelial differentiation status and mediates MET function in breast cancer cells

**Figure 2:**
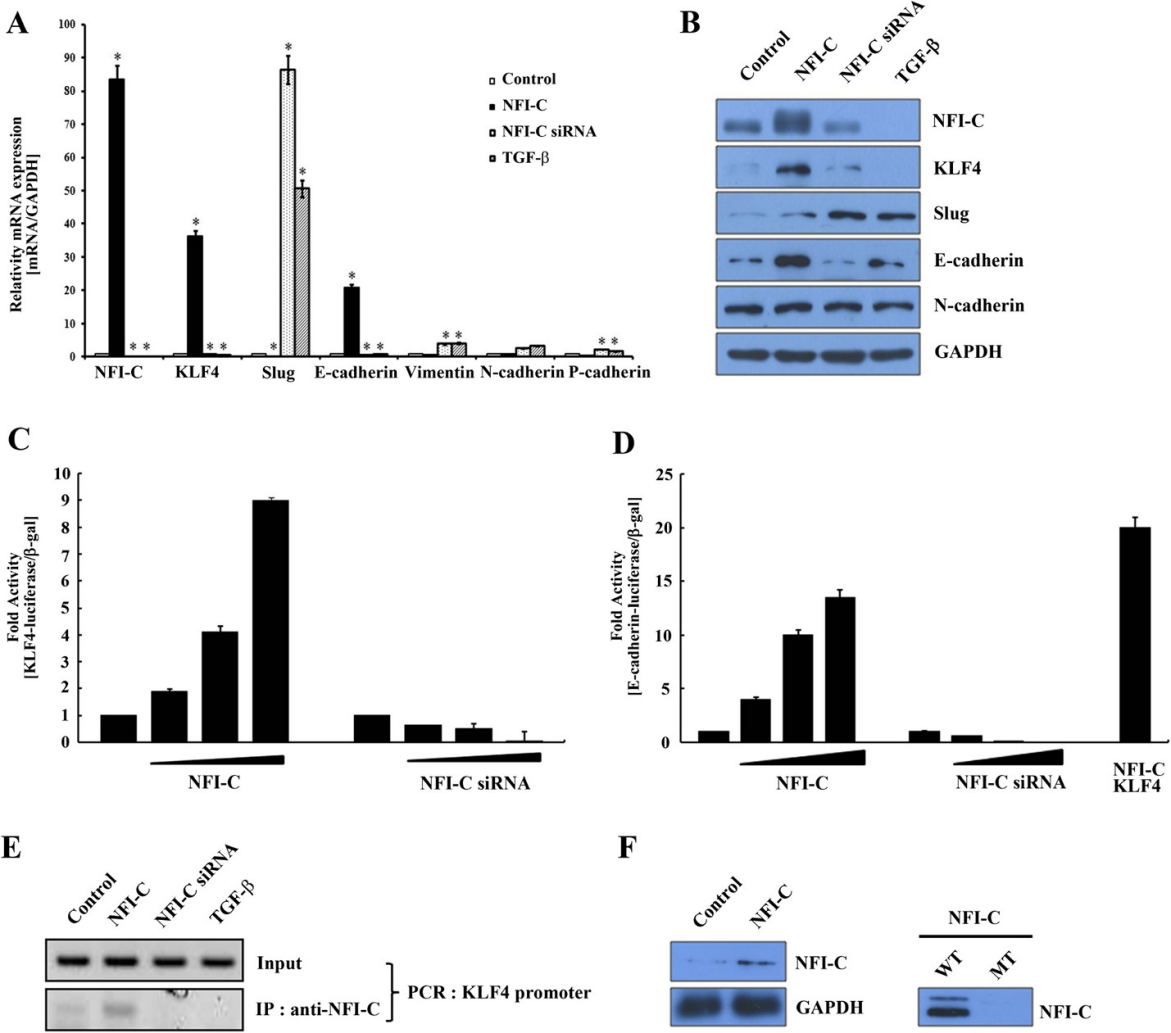
**Effects of NFI-C overexpression and inactivation on the KLF4-E-cadherin signaling pathway in MCF7 breast cancer cells. (A, B)** Expression of *NFI-C*, *KLF4*, *Slug*, *E-cadherin*, *Vimentin*, *N-cadherin*, and *P-cadherin* was analyzed by real-time PCR **(A)** and western blotting **(B)** in MCF7 cells transfected with *NFI-C*-expressing or *NFI-C*-siRNA constructs or treated with TGF-β. **(C, D)** Transcriptional activity of the *KLF4* promoter and the *E-cadherin* promoter were evaluated by luciferase assays using *NFI-C*-expressing or *NFI-C*-siRNA constructs in MCF7 cells. Control samples were transfected with only *E-cadherin* promoter constructs. Data are presented as the mean ± SD of triplicate experiments. * denotes values significantly different from the control (*P* < 0.01). **(E)** NFI-C binding to the *KLF4* promoter was investigated using ChIP assays after transfection with *NFI-C*-expressing or *NFI-C*-siRNA constructs in MCF7 cells. **(F)** The NFI-C binding motif in the *KLF4* promoter was confirmed by DNAP assay using extracts from MCF7 cells that had been transfected with *NFI-C-*expressing or *NFI-C*-siRNA constructs. Biotinylated oligonucleotides corresponding to the *KLF4* promoter (WT or mutated) were used as probes. WT, wild type; MT, mutant type.

MET initiates and completes the invasion-metastasis cascade of cancer cells [24]. Our previous results showed that NFI-C induces MET during normal odontoblast differentiation [17]. Therefore, we speculated that NFI-C also regulates the balance between KLF4 and E-cadherin in breast cancer cells, which is important for MET. To address this, we examined whether NFI-C could regulate the transcription of KLF4 and E-cadherin and subsequently alter the expression of marker genes in MCF7 breast cancer cells. MCF7 cells were transfected with *NFI-C*-expressing or *NFI-C*-siRNA constructs or treated with TGF-β. *NFI-C* overexpression increased the transcription of *KLF4* and *E-cadherin* mRNAs, which play an important role in the MET of cancer cells. In contrast, siRNA or TGF-β-mediated silencing of *NFI-C* decreased transcription of *KLF4* and *E-cadherin*. However, *NFI-C* expression levels did not significantly affect the expression of *N-cadherin* compared with other mRNA (Figure 2A). Slug (Snail2), a Snail family member, is the dominant regulator of EMT initiation *in vitro* and *in vivo*, as EMT is inhibited following Slu*g* depletion [25]. KLF4 and another epithelial determinant, FoxA1, are direct transcriptional inhibitors of Slug expression in mouse and human prostate cancer cells [26]. Interestingly, similar with microarray data with pulp cells of *Nfic* knockout mice [17], *Slug* mRNA was increased in *NFI-C* downregulated cells (Figure 2A). Also, *Vimentin* and *P-cadherin* mRNA was slightly increased in *NFI-C* downregulated cells by siRNA or TGF-β (Figure 2A). In western blot analyses, although *NFI-C* overexpression did not alter the expression levels of Slug and N-cadherin, it enhanced the expression of KLF4 and E-cadherin. Conversely, *NFI-C* inactivation by siRNA suppressed E-cadherin expression and induced Slug expression (Figure 2B). In addition, increasing the concentration of *NFI-C* significantly enhanced the expression of luciferase reporter genes under the control of the mouse *KLF4* promoter. In contrast, depletion of *NFI-C* using a specific siRNA suppressed promoter activity of the *KLF4* reporter construct in MCF7 cells (Figure 2C). *NFI-C* overexpression augmented *E-cadherin* promoter activity; whereas, siRNA-mediated *NFI-C* inactivation decreased *E-cadherin* promoter activity (Figure 2D). Furthermore, overexpression of *NFI-C* and *KLF4* showed a synergistic effect on *E-cadherin* transcription levels (Figure 2D).

In previous paper, Nfic binds to *Klf4* promoter but not to *Klf4* promoter with the mutation of Nfic binding site [17]. To confirm this data in tumor cells, we performed ChIP assay with the primer of *KLF4* promoter. The *KLF4* promoter could be precipitated using an NFI-C-specific antibody in *NFI-C*-expressing cells but not in *NFI-C*-silenced cells (Figure 2E). Similar with ChIP assay, DNAP assay showed that the wild type of the *KLF4* promoter bound with NFI-C protein, but not NFI-C proteins with mutations in these regions (Figure 2F). These studies suggest that NFI-C regulates E-cadherin expression by controlling the *KLF4* promoter in cancer cells.

### NFI-C suppresses EMT, migration, and invasion in breast cancer cells

**Figure 3:**
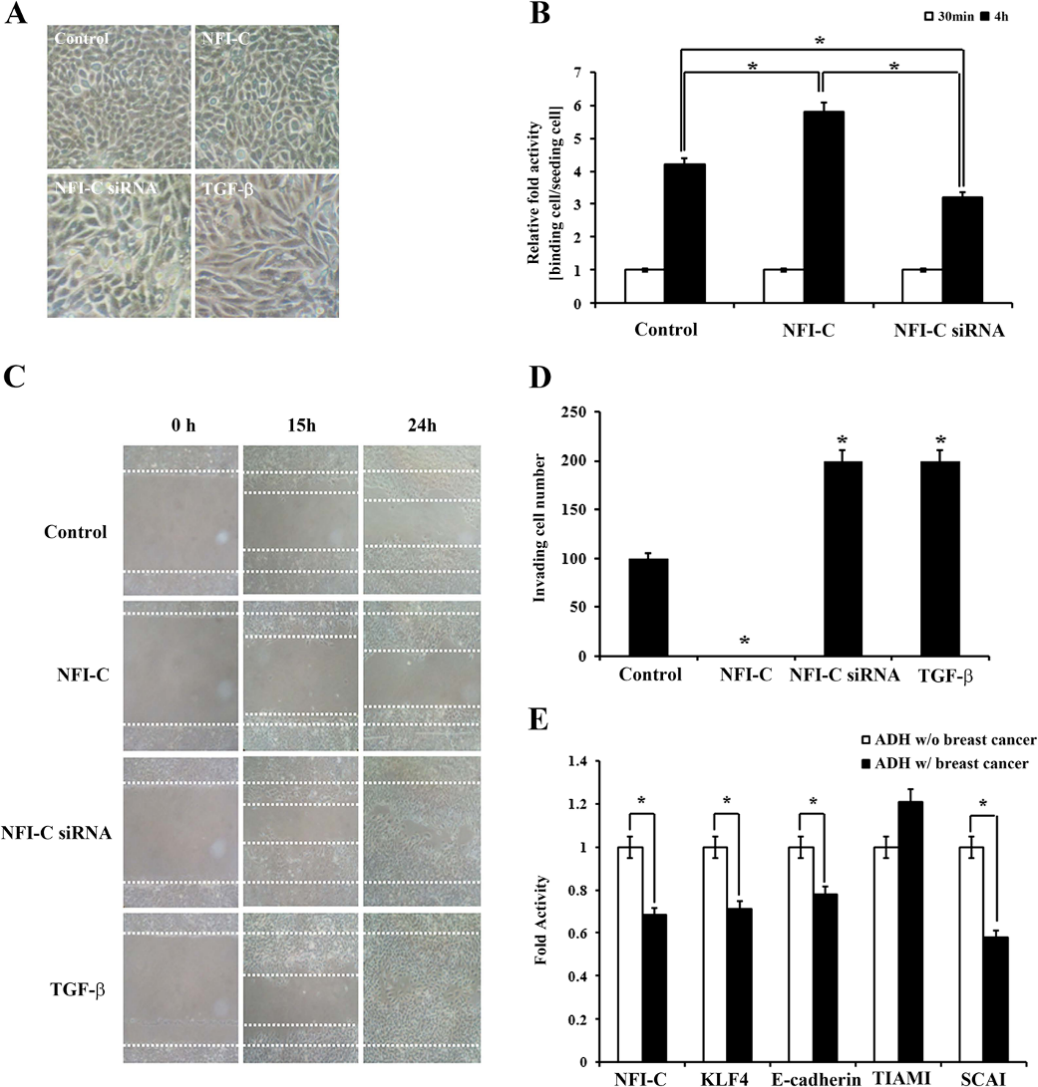
**Effects of NFI-C overexpression and inactivation on morphology, adhesion, migration, and invasion of breast cancer cells. (A)** Morphology of MCF10A cells when transfected with *NFI-C*-expressing or *NFI-C*-siRNA constructs, or treated with TGF-β. Images obtained using phase-contrast microscopy (Magnification: 100×). **(B)** Cell adhesion was assessed in MCF7 cells transfected with *NFI-C*-expressing or *NFI-C*-siRNA constructs. Data are presented as the mean ± SD of triplicate experiments. **(C)** Migration was analyzed by wound healing assays in MCF7 cell transfected with *NFI-C*-expressing or *NFI-C*-siRNA constructs or treated with TGF-β (Magnification: 400×). **(D)** The invasion capacity of MCF7 cells, which were transfected with *NFI-C*-expressing or *NFI-C*-siRNA constructs, or treated with TGF-β was determined by matrigel-coated transwell assays. Average cell counts from representative fields for each condition are given as mean ± S.D. **(E)** The effect of NFI-C on EMT of breast cancer cells was analyzed using gene expression data collected from atypical ductal hyperplasia with or without breast cancer in the Gene Expression Omnibus (GEO) database (GSE 2429). The mean and standard variants were calculated from four biological replicates for both activity and mRNA levels of *NFI-C*, *KLF4*, *E-cadherin*, *TIAM1* (invasion activator), and *SCAI* (invasion suppressor). * denotes values significantly different from the control (*P* < 0.01). ADH: Atypical ductal hyperplasia.

It is well-known that forced expression of TGF-β induces EMT [27]. Breast cancer cell lines range from epithelial-like with low invasiveness, to mesenchymal-like, which exhibit high invasive capacity [28]. Therefore, we first investigated whether NFI-C expression levels correlate with cellular phenotype in natural isolation. *NFI-C-*expressing MCF10A cells grew as tightly packed patches of epithelial sheets like normal MCF10A cells. In contrast, *NFI-C*-siRNA-transfected or TGF-β-treated MCF10A cells were longer, like mesenchymal cells (Figure 3A). In cell binding assays, NFI-C*-*expressing MCF7 cells showed significantly greater attachment than normal MCF7 cells on plates (Figure 3B). Next, we asked whether NFI-C controlled migration and invasion of breast cancer cells by MET. In wound healing assays, *NFI-C* enhanced MCF7 breast cancer cell migration. Conversely, ectopic *NFI-C*-siRNA or TGF-β treatment reduced MCF7 cell motility (Figure 3C). Transwell invasion assays also demonstrated a significantly decreased number of invasive MCF7 cells when transfected with *NFI-C*-expressing constructs compared with control cells, *NFI-C*-siRNA-transfected cells, or TGF-β-treated cells (Figure 3D). These results indicated that NFI-C is crucial for the inhibition of breast cancer cell migration and invasion *in vitro*.

In cancer cells, we analyzed the invasion-related expression of NFI-C, KLF4, and E-cadherin using GEO data. NFI*-*C, KLF4, and E-cadherin expression decreased in atypical ductal hyperplasia with breast cancer cells compared with atypical ductal hyperplasia without breast cancer cells. Furthermore, we examined genes that are related to metastasis and invasion such as *TIAM*1 and *SCAI*. Expression of TIAM1, an invasion factor, increased; whereas, expression of *SCAI,* a suppressor of invasion, decreased in atypical ductal hyperplasia with breast cancer cells compared with atypical ductal hyperplasia without breast cancer cells (Figure 3E). Therefore, NFI-C is an inducing target of the KLF4/E-cadherin pathway and plays a significant role in EMT repression.

### NFI-C and NFI-X influence KLF4 transcription

**Figure 4:**
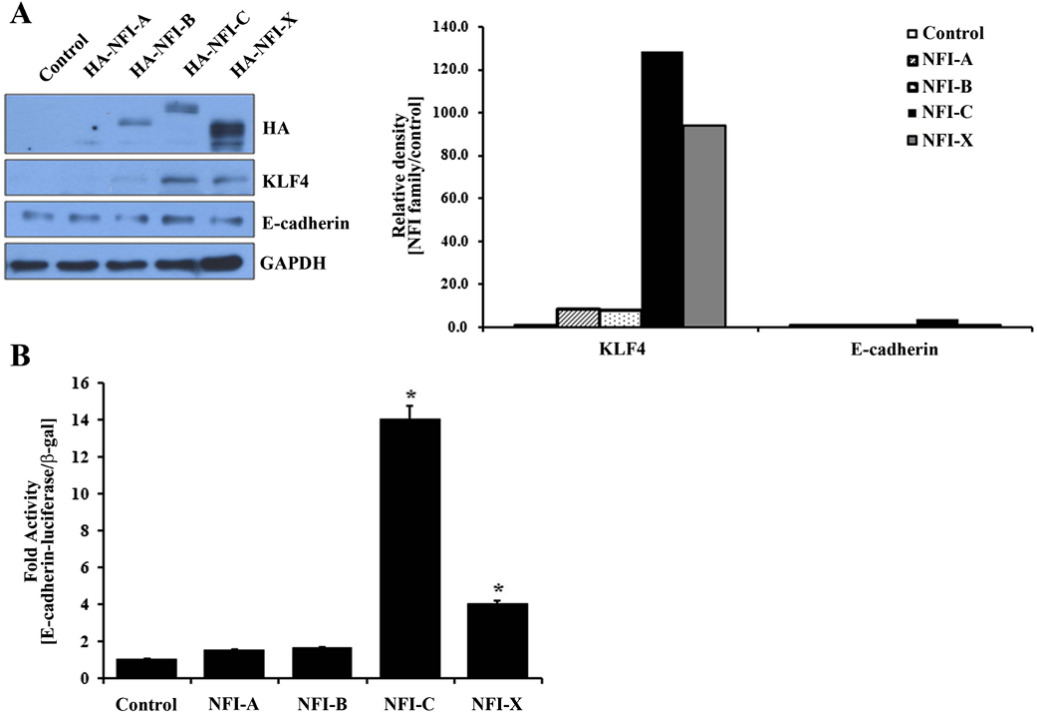
**Effects of NFI family members on the expression of KLF4 and E-cadherin. (A)** Expression of HA, KLF4, and E-cadherin proteins was analyzed by western blotting (left panel) and quantified (right panel) in MCF7 cells transfected with HA-tagged-*NFI-A*, −*NFI-B*, −*NFI-C*, or -*NFI-X* expressing constructs. **(B)** The transcriptional activity of the *E-cadherin* promoters was evaluated by luciferase assay in MCF7 cells transfected with *NFI-A*-, *NFI-B*-, *NFI-C*-, or *NFI-X*-expressing constructs. The data are presented as the mean ± SD of triplicate experiments. * denotes values significantly different from the control (*P* < 0.01).

NFI family members inhibit tumorigenesis by regulating oncogenic transcriptional factors such as brain fatty acid-binding protein (B-FABP) and JUN proteins [29-31]. To investigate the function of the four NFI factors on EMT/MET regulation, we examined the effects of NFI family member overexpression on *KLF4* and *E-cadherin* promoter activity. Expression of each of the NFI isoforms was confirmed by western blotting with the anti-HA antibody after transfection. Overexpression of *NFI-C* and *NFI-X* resulted in a statistically significant increase in *KLF4* and *E-cadherin* transcriptional activity in MCF7 breast cancer cells, while *NFI-A* and *NFI-B* overexpression did not (Figure 4).

Next, expression vectors encoding *NFI-A*, *NFI-B*, *NFI-C*, or *NFI-X* were co-transfected with *E-cadherin* reporter constructs into MCF7 cells. Transfection of *NFI-C* and *NFI-X* constructs significantly enhanced *KLF4* and *E-cadherin* promoter activity, suggesting that these two isoforms function as transcriptional activators of *KLF4* and *E-cadherin*. In contrast, transfection with *NFI-A* or *NFI-B*–expressing constructs did not noticeably activate *KLF4* or *E-cadherin* (Figure 4B).

### NFI-C and E-cadherin were strongly immunostained in normal human breast tissue

**Figure 5:**
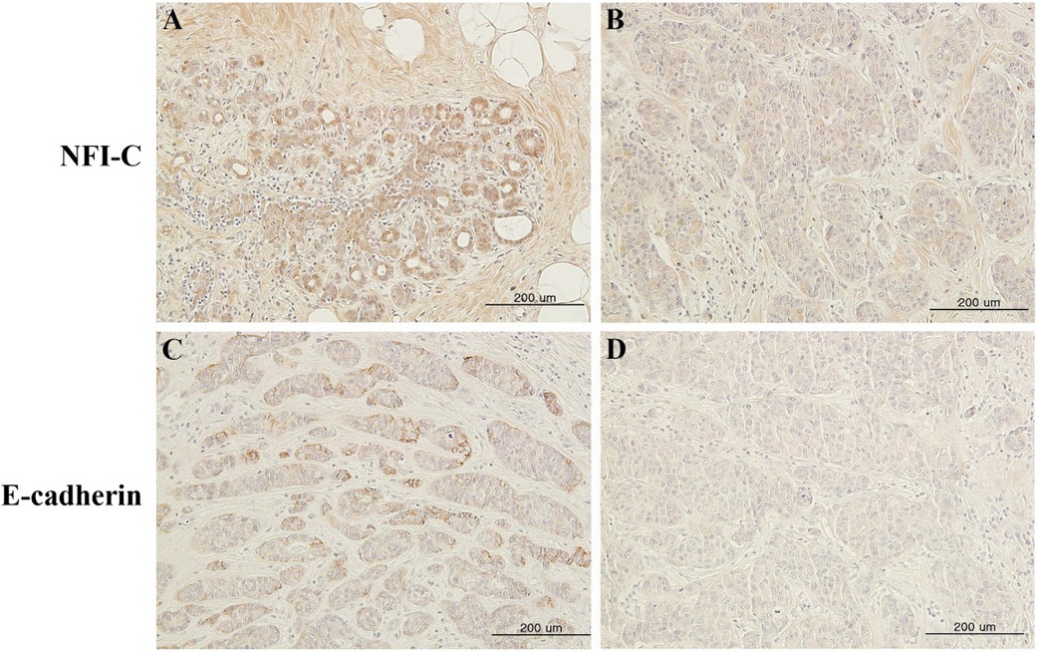
**Expression of NFI-C and E-cadherin protein in malignant human breast tissue by immunohistochemistry. (A)** Expression of NFI-C in a benign breast ductile. **(B)** Expression of NFI-C in a malignant ductal carcinoma. **(C)** Expression of E-cadherin in early stage tumor cells. **(D)** Expression of E-cadherin in a malignant ductal carcinoma. Scale bars = 200 μm.

NFI-C was stained stronger in normal glandular cells than tumor cells [14]. To assess the expression pattern of NFI-C and E-cadherin in breast tumors, we examined the NFI-C and E-cadherin protein expression in human breast cancer. Normal breast cells and early stage tumor cells uniformly stained strongly positive for NFI-C and E-cadherin protein (Figure 5A, C). In contrast, malignant ductal carcinoma cells revealed weak positive staining for anti-NFI-C and -E-cadherin (Figure 5B, D). These results suggested that NFI-C is an important factor for the epithelial character *in vivo* in that NFI-C and E-cadherin is expressed in normal human breast tissues, but its expression is reduced in malignant human breast tumors.

## Discussion

Unlike epithelial cells, which are stationary and characterized by apical-basal polarity, tight junctions, and expression of cell-cell adhesion markers such as E-cadherin, mesenchymal cells do not make mature cell-cell contacts, can invade through the ECM, and express markers such as Vimentin, Fibronectin, N-cadherin, Twist, and Snail [32,33]. Moreover, induced pluripotent stem cells (iPSCs) are derived from mouse embryonic fibroblasts (MEFs) by MET at the early stage of reprogramming [34]. These results suggest that MET is associated with normal development, cancer metastasis, inhibiting cancer progression, stem cell generation, and induced pluripotent stem cell reprogramming. However, relatively little is known regarding MET, compared to the extensive studies of EMT in dental organ development and tumor metastasis.

Recently, researchers have investigated MET pathways as potential therapeutic targets in organ regeneration and the prevention of metastases [35]. TGF-β, an important inducer of EMT, degrades NFI-C, whereas NFI-C induces dephosphorylation of p-Smad2/3, a TGF-β signaling molecule, in odontoblasts and breast cancer cells [16]. Thus, NFI-C counteracts EMT, motility, invasiveness, and tumor growth [14]. KLF4, an important inducer of MET, activates the epithelial program by triggering E-cadherin expression and induces MET in normal mammary epithelial cells and breast cancer cells [35].

E-cadherin and N-cadherin, both expressed in pulp cells during odontogenesis, are involved in the regulation of various biological processes, such as cell recognition, intercellular communication, cell fate, cell polarity, boundary formation, and morphogenesis [36,37]. In *NFI-C*^−/−^ mice, *KLF4* mRNA expression is significantly decreased compared to wild-type cells. *NFI-C* binds directly to the *KLF4* promoter and stimulates *KLF4* transcriptional activity, thereby regulating Dmp1 and DSPP expression during odontoblast differentiation, and then promoting mineralized nodule formation in MDPC-23 cells. It is involved with important signaling pathways during dentinogenesis: the NFI-C-KLF4-Dmp1-Dspp and the NFI-C-KLF4-E-cadherin pathways in odontoblasts [17]. Consistent with these observations, odontoblastic MDPC-23 cells show opposite patterns of NFI-C/E-cadherin and TGF-β/N-cadherin expression [16,17].

In the present study, NFI-C was shown to regulate KLF4, a MET inducer, and subsequently controlled E-cadherin expression in normal mammary epithelial cells (MCF10A) and breast cancer cells (MCF7). Consistent with previous investigations of odontoblasts, NFI-C also induced MET in breast cancer cells via upregulation of *KLF4* and *E-cadherin* and down-regulation of *Slug*, a dominant regulator of EMT initiation. NFI-C suppressed migration and invasion in breast cancer cells. In contrast, *NFI-C* inactivation by siRNA promoted breast cancer cell migration and invasion. These results suggest that NFI-C induces MET in cell types other than odontoblasts, such as cancer cells, through regulation of the *KLF4*-*E-cadherin* axis, thus preventing cancer cell migration and invasion.

*NFI* family members (*NFI-A*, *NFI-B*, *NFI-C*, and *NFI-X*) exhibit promoter-specific differences in their maximal transcriptional activation potentials due to differences in their carboxy-terminal regions [38]. NFI proteins exhibit cell type- and promoter-specific differences in their repression properties, with NFI-C and NFI-X, but not NFI-a and NFI-B, repressing the mouse mammary tumor virus (MMTV) promoter in HeLa cells. NFI-C-mediated repression occurs by interference with coactivator (e.g., p300/CBP or SRC-1A) function at the MMTV promoter [30]. Although it is widely accepted that NFI proteins differ in their repression and activation potentials, their ability to regulate transcription is poorly understood. In the present study, *NFI-C* and *NFI-X* influenced *KLF4* transcription but *NFI-A* and *NFI-B* did not. *NFI-C* and *NFI-X* likely regulate *KLF4* because the DNA-binding affinities of NFI-C and NFI-X were higher than those of NFI-a and NFI-B; however, no direct evidence supports this notion.

## Conclusions

**Figure 6:**
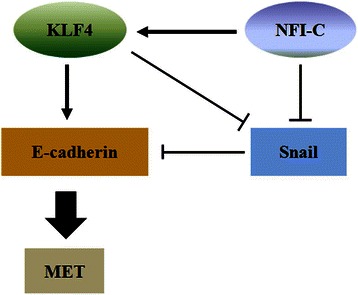
**Model for the function of NFI-C and KLF4 during tumorigenesis.**

As summarized in Figure 6, NFI-C was strongly expressed in normal mammary gland cells. NFI-C increased the expression of KLF4 and E-cadherin, and led to a more pronounced epithelial cell phenotype. In contrast, NFI-C knock-down induced migration and invasion. These results demonstrate that NFI-C is essential for the maintenance of epithelial differentiation and is required to reduce EMT and metastasis by regulation of KLF4 and E-cadherin expression. This work is the first to investigate the NFI-C-KLF4-E-cadherin signaling pathway in breast cancer cells, as well as their functional implications during tumorigenesis. This information will lead to a comprehensive understanding of the role of NFI-C in cancer.

## Abbreviations

NFI-C: Nuclear factor I-C
KLF4: Krüppel-like factor 4
DSPP: Dentin sialophosphoprotein
DMP1: Dentin matrix protein 1
